# Feasibility of deformation-independent tumor-tracking radiotherapy during respiration

**DOI:** 10.4103/0971-6203.79691

**Published:** 2011

**Authors:** Seonkyu Kim, Myonggeun Yoon, Dong Ho Shin, Dongwook Kim, Sangyeob Lee, Se Byeong Lee, Sung Yong Park, Sang Hyuk Song

**Affiliations:** Proton Therapy Center, National Cancer Center, Goyang, Korea; 1 Seoul National University Hospital, Seoul, Korea

**Keywords:** Deformation, real-time tumor tracking, respiration, 4D-CT

## Abstract

To evaluate the feasibility of tumor-tracking radiotherapy that does not consider tumor deformation during respiration. Four-dimensional computed tomography (4D-CT) data, which considers 10 phases of the respiration cycle, were acquired in 4 patients with lung cancer and 4 patients with liver cancer. Initial treatment plans were established at the end of the inhalation phase (phase 1). As a simulation of deformation-free tumor-tracking radiotherapy, the beam center of the initial plan was moved to the tumor center for all other phases, and the tumor shape acquired from phase 1 was used for all 10 phases. The feasibility of this method was analyzed based on assessment of equivalent uniform dose (EUD), homogeneity index *(HI)* and coverage index (COV). In photon radiation treatment, movement-induced dose reduction was not particularly significant, with 0.5%, 17.3% and 2.8% average variation in EUD, *HI* and COV, respectively. In proton radiation treatment, movement-induced dose reduction was more significant, with 0.3%, 40.5% and 2.2% average variation in EUD, *HI* and COV, respectively. Proton treatment is more sensitive to tumor movement than is photon treatment, and that it is reasonable to disregard tumor deformation during photon therapy employing tumor-tracking radiotherapy.

## Introduction

The objective of radiation therapy is to administer radiation to a tumor, with minimization of radiation received by adjacent normal tissues. Treatment planning is used to accurately target a tumor and to optimize the administration of radiation, which is typically three-dimensional conformal radiation therapy (3D-CRT) or intensity-modulated radiation therapy (IMRT). 3D-CRT and IMRT require high accuracy, because a multileaf collimator (MLC) must be shaped to match the tumor.[1,2] The shape of MLC is based on stationary medical images, such as those provided by computed tomography (CT), magnetic resonance imaging (MRI) and positron emission tomography (PET). Patient respiration or movement can change the location and shape of a tumor in a lung or liver. To compensate for these changes, the tumor margin is generally considered during treatment planning. For example, liver cancer has a setup margin of at least 1.5 cm during respiration, and is considered in planning for the target margin.[3]

Various techniques have been developed to solve the intra-fractional motion of tumors, including the motion-encompassing method, holding of breath, respiratory gating, and real-time tumor-tracking. The motion-encompassing method requires an estimation of the position and range of tumor motion during respiration, as provided by 4D-CT.[4–6] However, if patient respiration is irregular, tumor size may be overestimated, and the radiation dose may be unnecessarily high. In the breath-holding method, the patient holds his/ her breath, and the position of the patient is maintained in all fractions. In the forced-shallow breathing method, respiration movements are limited by a physical plate positioned on the abdominal region.[7,8] However, this procedure may result in significant patient discomfort and poor reproducibility.

Several research centers are studying the use of respiratory gating to deal with respiratory motion during radiotherapy of thoracic and abdominal tumors.[9] This procedure is essentially the same as 3-D conformal therapy. In particular, imaging and treatment are synchronized with the patient’s respiratory cycle, thereby reducing the margin of difference between the clinical target volume (CTV) and the planning target volume (PTV).[10] Respiratory-gating methods are categorized as internal or external, depending on the use of surrogates. During the respiratory cycle, the position and width of the gate are obtained by monitoring the respiratory motion of the patient using an external signal and a fiducial marker. External gating uses a surrogate marker (typically on the patient’s abdomen), and internal gating employs an implanted marker. A drawback of this method is that marker and tumor motion may be different. In addition, the relationship between tumor motion and the surrogate signal is unstable and can vary over time.

Real-time tumor-tracking systems provide a method for reducing the effect of respiration-induced target motion. Such tumor-tracking using an MLC (multileaf collimator) has been introduced in previous studies; it allows easy adjustment of the size and shape of the irradiating photon beam.[11,12] The simplest approach is to move the collimator. However, in tumor-tracking radiotherapy, it is essential to know the exact tumor location and shape. The location of a tumor can be tracked using various methods, but real-time tracking of tumor shape *cannot* be achieved by the 2D imaging systems that are conventionally used in treatment rooms.

There have been many studies of real-time tumor-tracking, but little is known about the effect of radiation dose when changes in tumor shape are ignored during tumor-tracking radiotherapy. In the present study, we evaluated the feasibility of tumor-tracking radiotherapy used without consideration of tumor deformation.

## Materials and Methods

### 

#### Patient characteristics and treatment planning

**Figure 1:**
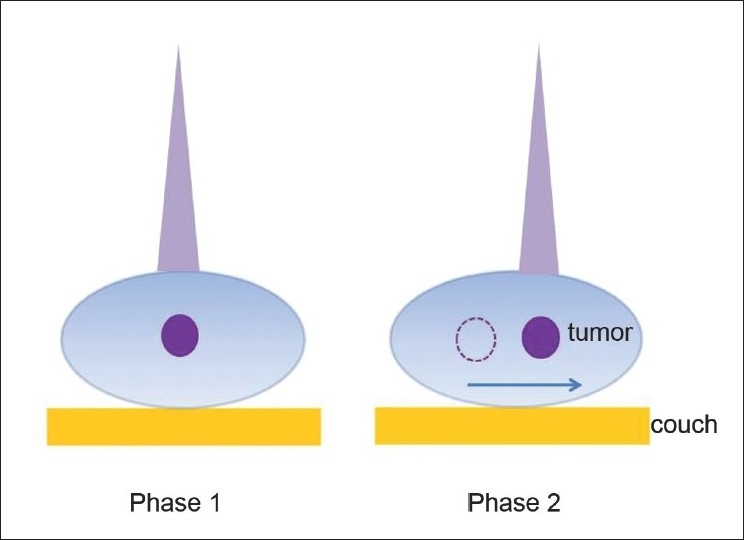
Schematic picture for tumor-tracking simulation. The beam center is moved to the tumor center based on the location of moving tumor

Four lung cancer patients and 4 liver cancer patients were analyzed at our institution using 4D-CT, which divided the respiratory cycle into 10 phases. Phase 1 corresponds to the end of an inhalation phase (0%). Figure 1 shows a schematic of our tumor-tracking simulation. This figure shows that the beam center of the initial plan was moved to the tumor center in other phases, using tumor shape (considered to be constant) acquired from phase 1. The targets were defined in accordance with the report of the International Commission on Radiation Units and Measurements (ICRU 50). In particular, gross tumor volume (GTV) encompassed all detectable tumors and lymph nodes as observed on CT scans. Planning target volume (PTV) included the GTV plus a 10-15–mm margin. To be consistent for tumor contouring, the same clinician manually drew all the tumor volumes in the 10 phases of CT.

Table 1 lists the clinically relevant patient characteristics and treatment schemes. All treatment plans for lung and liver cancer patients used 3 to 5 beams for IMRT and 2 to 3 beams for Proton beam therapy (PBT). The proximal, distal and transverse margins were 2, 2 and 10 mm, respectively; and the border smoothing and smearing margins were set at 0 and 3 mm, respectively. We used relatively small margins in planning, because various uncertainties had already been included in our PTV.

**Table 1:** Patient characteristics and treatment protocols

*Primary pathology*	*Modality*	*Gender*	*Age (years)*	*Fractional dose (Gy)*	*Number of fractions*	*Prescribed dose (Gy)*
Hepatocellular carcinoma	Proton	M	70	2.5	20	50.0
Hepatocellular carcinoma	Proton	M	56	2.5	22	55.0
Hepatocellular carcinoma	Proton	F	70	2.5	22	55.0
Hepatocellular carcinoma	Proton	F	56	2.5	24	60.0
Squamous cell carcinoma	Photon	M	77	2.2	30	66.0
Squamous cell carcinoma	Photon	M	69	2.4	27	64.8
Squamous cell carcinoma	Photon	M	78	2.4	30	72.0
Squamous cell carcinoma	Proton	M	77	6.0	10	60.0

#### Homogeneity index

Adequate assessment of the homogeneity of target volume is important for calculating the dose volume histogram (DVH). Losses in tumor-control probability will occur for cold-spot–induced dose inhomogeneity, so it is essential to properly evaluate the homogeneity of the target volume. The homogeneity index *(HI)* is defined as[13]

(1) $$HI\;=\;\frac{D_{2}–D_{98}}{D_{P}}\times 100\%$$

where *D*_2_ and *D*_98_ represent the doses to 2% and 98% of the target volume, respectively, and *D_p_* is the prescribed dose. *D*_98_, which may be considered the minimum dose, is the dose received by 98% of the target volume; D_2_, which may be considered the maximum dose, is the dose received by 2% of the target volume. A lower *HI* indicates a more homogeneous target dose. The normalized *HI* (n*HI*) is the ratio of the *HI* of the shifted target to the *HI* of the original target.

#### Coverage index

In addition to providing information on the homogeneity of radiation doses, DVHs can also be used to assess target coverage based on the coverage index *(COV)*, defined as the percentage of tumor volume that received the prescribed dose. Ideally, tumor DVH would be a step function, with 100% of the target receiving the exact prescribed dose. However, actual DVH curves are not step functions, because of constraints imposed by tumor volume and other organs at risk (OAR). The conditions for clinically acceptable target volume coverage include (i) no more than 20% of any planning target volume (PTV) will receive > 110% of the prescribed dose; (ii) the prescribed dose is the isodose that encompasses at least 95% of the PTV; and (iii) no more than 1% of any PTV will receive < 93% of the prescribed dose.[14] The last two conditions indicate that coverage indices at D_p_ and at 93% of D_p_ should be more than 95% and 99%, respectively. The normalized *COV* (n*COV*) is the ratio of the *COV* for the shifted target to the *COV* of the original target.

#### Equivalent uniform dose

When evaluating the dose homogeneity of a treatment plan, it is necessary to consider the radiobiological impact of dose inhomogeneity on target volume. The equivalent uniform dose (EUD) is one parameter that describes the relationship between homogeneity and radiobiological effect. The EUD is defined as the biologically equivalent dose that if given uniformly, would lead to the same reduction in tumor volume as the actual inhomogeneous dose distribution.[15] It is calculated as

(2), $$EUD=\frac{D_{ref}}{{\mathrm{lnSF}}_{2}}ln\left(\frac{\Sigma V_{i}\rho_{i}SF_{2}D_{i}/D_{ref}}{\Sigma V_{i}\rho_{i}}\right)$$

The reference dose (D*_ref_*) and surviving fraction (SF_2_) are typically set at 2 Gy and 0.5 Gy, respectively. Summation is performed over all bins of the DVH with a volume element (V*_i_*) and a clonogenic cell density (*r_i_*), both of which are uniform. EUD is calculated as the percentage of the prescribed dose, and normalized EUD (nEUD) is the ratio of the EUD for the shifted target to the EUD of the original target.

## Results

**Figure 2:**
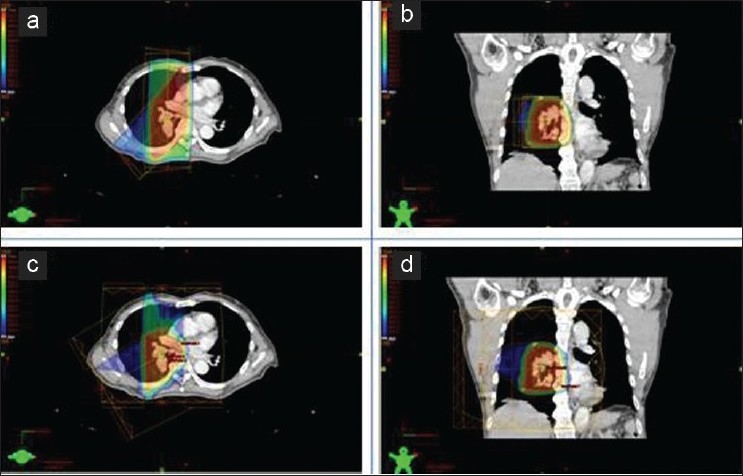
An example of a radiotherapy plan for treatment of lung cancer using photon and proton therapies. (a) Axial view, (b) coronal view in photon treatment; and (c) axial view, (d) coronal view in proton treatment are shown

**Figure 3:**
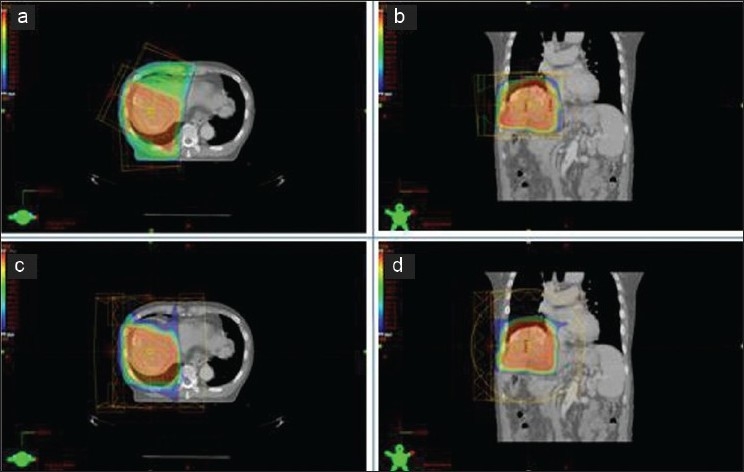
An example of a radiotherapy plan for treatment of liver cancer using photon and proton therapies. (a) Axial view, (b) coronal view in photon treatment; and (c) axial view, (d) coronal view in proton treatment are shown

Figure 2 shows an example of a radiotherapy plan for treatment of lung cancer using photon and proton therapies. The dose map for lung cancer proton therapy [Figures 2c and 2d] is clearly better than that for photon therapy [Figures 2a and 2b]. Similarly, the treatment plans for liver cancer show better dosimetric behavior of proton therapy compared with photon therapy [Figure 3]. All treatment plans were established with a CT set during phase 1, and satisfy conventional dosimetric factors (dose conformity, dose homogeneity and dose coverage).

**Figure 4:**
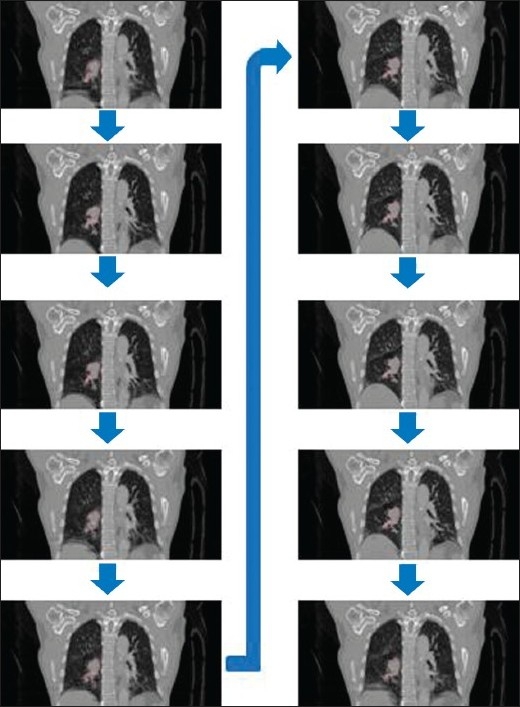
Coronal slices depicting the peak-inhale phase (phase 1, top left), peak-exhale phases (phases 5-6, bottom left and top right), peak-inhale phase (phase 10, bottom right) and a few others of a 4-D computed tomography (4D CT) scan. The contour represents the tumor volume as delineated by the physician on the planning CT

Figure 4 shows an example of 10 coronal images of the 10 phases of respiration in 4D-CT. This figure shows the relationship between tumor shape and location with respect to respiration phase. Although it is not particularly clear, tumor shape definitely changes throughout the 10 phases, with a change in position of up to 2-3 cm. This suggests that dosimetric factors may vary because of tumor motion during radiation therapy.

Table 2 shows variation in tumor size during the 10 respiratory phases. Average tumor size varied by up to ~8%, suggesting that volume changes during respiration may need to be considered. Tumor size is largest in phases 5 to 7.

**Table 2:** Planning target volume (relative to phase 1) during 10 phases of respiration

*Phase*	*Relative planning target volume*			
	*Lung*		*Liver*	
	*Mean*	*STDEV*	*Mean*	*STDEV*
Phase 1	100.0	0.0	100.0	0.0
Phase 2	99.2	2.5	103.3	0.2
Phase 3	101.6	2.1	101.0	4.3
Phase 4	100.3	7.9	103.8	2.0
Phase 5	104.8	7.3	107.9	1.5
Phase 6	108.1	6.4	108.3	2.8
Phase 7	108.9	6.3	105.3	0.8
Phase 8	103.5	2.8	106.3	1.3
Phase 9	102.4	5.3	105.6	4.4
Phase 10	103.0	2.9	103.5	4.3

STDEV: Standard deviation

**Figure 5:**
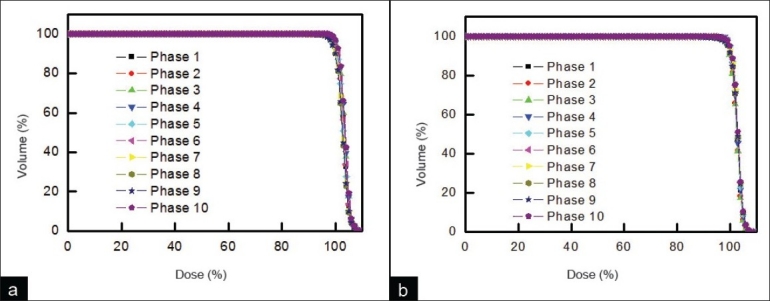
An example of dosimetric variation of DVH for (a) photon treatment and (b) proton treatment of 1 lung cancer patient when tumor-tracking radiotherapy was performed, assuming constant tumor shape (acquired from phase 1)

**Figure 6:**
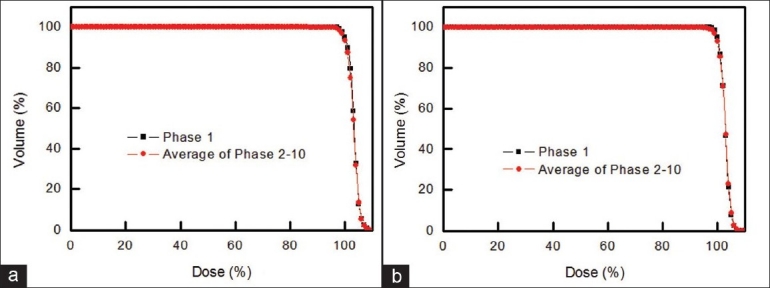
DVH for phase 1 and the average DVH of phases 2 to 10 for (a) photon treatment and (b) proton treatment of lung cancer patient seen in Fig. 5, which are similar, indicating that the same level of DVH can be acquired based on the proposed method

Figure 5 shows an example of dosimetric variation of DVH for photon and proton treatment of 1 lung cancer patient. DVHs were assessed when tumor-tracking radiotherapy was performed, assuming constant tumor shape (acquired from phase 1). In photon therapy [Figure 5a], DVH changed very little, suggesting that tumor-tracking radiotherapy without consideration of tumor deformation may be effective in photon therapy [Figure 5a]. DVH values for proton therapy [Figure 5b] also show little variation from the phase-1 DVH, suggesting that this method may also be suitable for proton therapy. Figure 6 shows the DVH for phase 1 and the average DVH of phases 2 to 10 for photon and proton treatments of lung cancer patient seen in Figure 5. Clearly, the DVH of phase 1 and the average DVH of phases 2 to 10 are similar, suggesting that tumor-tracking radiotherapy may be successful if lung tumor deformation is ignored.

**Figure 7:**
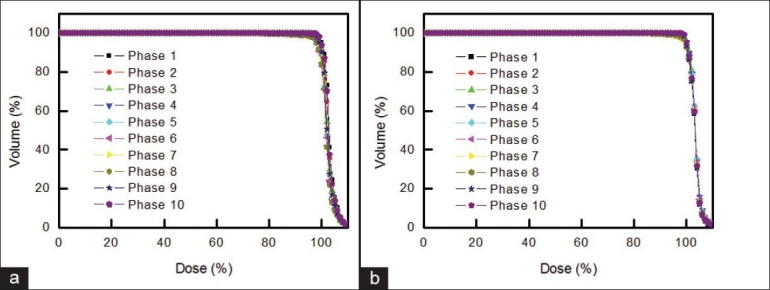
An example of dosimetric variation of DVH for (a) photon treatment and (b) proton treatment of 1 liver cancer patient when tumor-tracking radiotherapy was performed, assuming constant tumor shape (acquired from phase 1)

**Figure 8:**
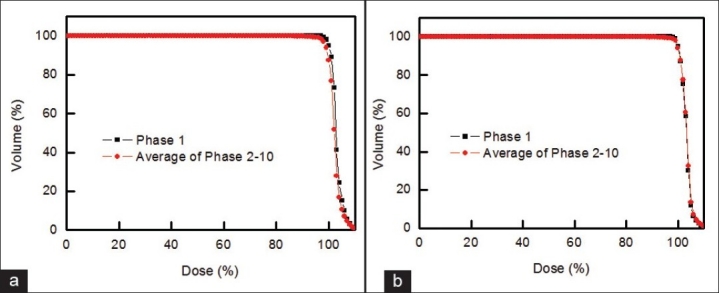
DVH for phase 1 and the average DVH of phases 2 to 10 for (a) photon treatment and (b) proton treatment of liver cancer patient seen in Figure 7, which are similar, indicating that the same level of DVH can be acquired based on the proposed method

Figure 7 shows an example of dosimetric variation of DVH for photon and proton treatments of 1 liver cancer patient. The results are similar to those for lung cancer [Figure 5], indicating that tumor deformation during respiration can also be ignored for radiotherapy of liver cancer. Figure 8 shows that the DVH of phase 1 and the average DVH of phases 2 to 10 are similar for liver cancer, as was the case with lung cancer [Figure 6].

Table 3 shows the averaged dosimetric changes during the 10 phases of respiration for lung and liver cancers together when tumor deformation is ignored. For photon therapy, the greatest change in minimum dose was 23.8% (during phase 6), but the average dose varied by only about 0.5%. For proton therapy, the greatest change in minimum dose was 38.6% (during phase 6), and average dose varied by about 1.0%.

**Table 3:** Radiation dose for the 10 phases of respiration, relative to phase 1; and the averaged data both for lung and liver cancers in photon and proton treatments

*Phase*	*Photon treatment*			*Proton treatment*		
	*Min*	*Max*	*Mean*	*Min*	*Max*	*Mean*
Phase 1	100	100	100	100	100	100
Phase 2	90.3	99.7	99.7	88.4	99.7	99.9
Phase 3	101.1	100.1	100	90.2	98.9	99.8
Phase 4	84	99.9	99.8	78.8	99.3	99.6
Phase 5	79.4	99.9	99.4	64.5	99.4	98.5
Phase 6	76.2	99.9	99.5	61.4	99.6	98.2
Phase 7	78.7	99.9	99.6	67.2	99.5	98.4
Phase 8	82.9	99.8	99.6	73.3	99.4	99.1
Phase 9	83.7	99.7	99.4	80.4	99.4	99.8
Phase 10	86.3	99.9	99.8	88.8	99.4	99.9

Min: Minimum, Max: Maximum, Mean: Mean

Finally, we calculated 3 dosimetric factors, nEUD, n*HI*, and nCOV [Table 4], to assess the role played by tumor movement during photon and proton therapies. In photon therapy, the average variation of nEUD, n*HI* and nCOV was 0.5%, 17.3% and 2.8%, respectively. In proton therapy, the average variation of nEUD, n*HI* and nCOV was 0.3%, 40.5% and 2.2%, respectively.

**Table 4:** Relative dosimetric factors for 10 phases compared to the factor for phase 1 in photon and proton treatments

*Phase*	*Photon treatment*			*Proton treatment*		
	*nEUD*	*nHI*	*nCOV*	*nEUD*	*nHI*	*nCOV*
Phase 1	100	100	100	100	100	100
Phase 2	99.3	107.1	96.9	99.8	106.7	99.2
Phase 3	99.8	101.5	99.3	99.9	102.7	99.6
Phase 4	99.7	117.7	97.2	99.7	139.4	98.1
Phase 5	99.1	140.8	95.6	99.6	191.6	96.9
Phase 6	99.2	131	96	99.5	190.1	95.3
Phase 7	99.4	126.1	96.4	99.6	171.4	96.3
Phase 8	99.4	121.4	96.3	99.7	144.6	96.2
Phase 9	99.2	119.1	95.9	99.7	140.8	97.3
Phase 10	99.5	108.2	98.1	99.8	117.6	98.7
Average	99.5	117.3	97.2	99.7	140.5	97.8

nEUD: Normalized EUD, nHI: Normalized HI, nCOV: Normalized COV respectively

## Discussion

In the present study, we examined the dosimetric effect of the radiation treatment plan during tumor-tracking radiotherapy, when tumor deformation is ignored. There have been numerous previous studies of tumor deformation during radiotherapy. In particular, Chhatkuli *et al*.[16] simulated the deformation of lungs and lung tumors during inspiration using a mesh-free simulation technique termed the *moving particle semi-implicit method* (MPS). The cited authors modeled deformation by considering lung tissues to be homogenous, isotropic and visco-elastic. The regional deformation of lung tumors with a superior-inferior (SI), right-left (RL) and anterior-posterior (AP) orientation was compared with experimental CT data taken at the end of inspiration. The authors compared their numerical results with experimental data obtained by tracking the movement of gold fiducial markers. Their results showed that deformation varied from less than 5 mm in the upper region to over 20 mm in the lower segment. For the tumor, however, both the experimental and numerical predictions showed that there was no volumetric change in tumor shape by the end of inspiration.

Thus, our experimental results with lung and liver cancer patients, along with previous simulation data on lung cancer, indicate that tumor volume and shape do not have significance throughout inspiration. However, both experimental results and numerical simulations indicate that the center point of a lung tumor was 5 mm lower (along the SI direction) at the end of inspiration. This suggests that tumor-center–tracking radiotherapy which assumes a constant tumor shape may be feasible and supports the proposal that tumor deformation during respiration may be disregarded in radiation therapy.

## Conclusion

A previous simulation study showed it was possible to establish a radiation treatment plan by tracking the movement of the tumor center, ignoring changes in tumor shape during respiration. Our experimental results with 4 lung cancer and 4 liver cancer patients suggest that proton therapy is more sensitive to tumor movement than is photon therapy, and that tumor deformation may be safely disregarded in tumor-tracking radiotherapy that employs photon therapy.
